# Optimizing surfactin yield in *Bacillus velezensis* BN to enhance biocontrol efficacy and rhizosphere colonization

**DOI:** 10.3389/fmicb.2025.1551436

**Published:** 2025-03-05

**Authors:** Tongshu Liu, Yanli Zheng, Litao Wang, Xu Wang, Haiyan Wang, Yongqiang Tian

**Affiliations:** School of Biological and Pharmaceutical Engineering, Lanzhou Jiaotong University, Lanzhou, China

**Keywords:** PGPR, surfactin, biocontrol, biofilms, colonization

## Abstract

**Introduction:**

Surfactins, a class of lipopeptide biosurfactants secreted by plant growth-promoting rhizobacteria (PGPR), have garnered significant attention due to their dual functionality in promoting plant growth and controlling plant diseases. Their potential as biopesticides is underscored by their unique physicochemical properties and biological activities. However, the practical application of surfactin is currently limited by its low yield in natural strains.

**Methods:**

This study aimed to optimize the culture conditions for *Bacillus velezensis* BN, a strain with exceptional biocontrol properties, to enhance its surfactin yield. Critical factors, including nitrogen sources and amino acid supplementation, were systematically investigated to determine their impact on surfactin production.

**Results:**

The study revealed that nitrogen sources and amino acid supplementation were pivotal factors influencing surfactin yield. Compared to the baseline, these factors resulted in a remarkable 5.94-fold increase in surfactin production. Furthermore, a positive correlation was established between surfactin yield and biocontrol efficacy. Enhanced surfactin yield was associated with improved antifungal activity, biofilm formation, and rhizosphere colonization capacity of *B. velezensis* BN on potato plantlets.

**Discussion:**

These findings provide novel insights into the practical application of surfactin and establish a scientific foundation for the development of innovative and eco-friendly antifungal agents suitable for agricultural use. The results demonstrate that optimizing culture conditions can significantly enhance surfactin yield and biocontrol efficacy, thereby highlighting the potential for sustainable agricultural practices.

## 1 Introduction

Agriculture is facing unprecedented challenges due to ecological damage caused by pest invasions, disease outbreaks, and the excessive use of chemical pesticides. While chemical pesticides provide effective short-term solutions for pest and disease control, their prolonged application often results in pathogen resistance and irreversible environmental damage (Hezakiel et al., 2024). In contrast, plant growth-promoting rhizobacteria (PGPR) present significant advantages as natural and renewable resources. PGPR can enhance plant resistance to diseases through mechanisms such as antibiotic production (Santoyo et al., 2021), suppression of pathogenic fungi growth (Ye et al., 2023), and promotion of plant growth (Babalola et al., 2024; Kondo et al., 2023), while minimizing environmental harm. Consequently, the development of eco-friendly alternatives to chemical pesticides is imperative for sustainable plant protection.

*Bacillus* species, as key members of PGPR, exhibit unique biological characteristics that enable them to produce a wide variety of secondary metabolites. These metabolites play critical roles in activating plant defense mechanisms, suppressing the growth of phytopathogens, and enhancing microbial colonization in the rhizosphere (Wu et al., 2024; Saleem et al., 2021). Among these metabolites, lipopeptides synthesized by non-ribosomal peptide synthetases (NRPS) have garnered significant attention due to their exceptional antimicrobial activity, thermal stability, and safety (Zhang and Zhou, 2022). These bioactive compounds have been widely utilized across diverse fields, including medicine (Deravel et al., 2014), petroleum technology (Kim et al., 2009), and agriculture (Munusamy et al., 2020).

Surfactin, a member of lipopeptides family with highly effective biosurfactant activity (Hu et al., 2019), is characterized by its amphiphilic structure, which consists of a hydrophilic cyclic peptide and a hydrophobic fatty acid chain linked via an ester bond (Dai et al., 2025). This unique structure imparts surfactin with exceptional biological surface activity and antimicrobial properties, making it a promising candidate for agricultural applications. Studies have demonstrated that surfactin can inhibit the growth of plant pathogens, enhance plant resistance (Xiao et al., 2023; Krishnan et al., 2019; Park et al., 2019), and promote the colonization of the production strain within plant tissues (Stoll et al., 2021; Kinsinger et al., 2003; Dong et al., 2022), which is essential for the biocontrol efficacy of PGPR, as the biocontrol role of PGPR is predicated on achieving a minimum threshold population density within the plant. Consequently, surfactin enhances the biocontrol capabilities of PGPR (Das and Dkhar, 2011). Given the low surfactin yield of natural strains, identifying PGPR strains with improved surfactin yield is crucial for the development of effective biocontrol strategies.

In recent years, the optimization of surfactin production by *Bacillus* species has garnered significant attention due to their considerable potential for practical applications. Research efforts worldwide have primarily focused on enhancing surfactin yield through optimization of fermentation conditions, adjustment of culture medium components, and application of response surface methodology (RSM). Zhou et al. (2019) reported an increase in surfactin production by *B. velezensis* BS-37d to nearly 2 g L^−1^ by supplementing the culture medium with glycerol and L-leucine. Similarly, Luo et al. (2023) achieved a 2.21-fold increase in surfactin yield to 8.37 g L^−1^ through medium optimization in *B. velezensis* BsC5. Atwa et al. (2013) enhanced surfactin production by *B. velezensis* NRC-1 through the optimization of a modified bench-top bioreactor. This innovative approach led to a significant increase in surfactin yield, from 6.3 g L^−1^ to 8.9 g L^−1^. These studies collectively demonstrate that rational optimization strategies can effectively improve surfactin production, highlighting the potential for further advancements in this area.

In this study, a strain of *Bacillus velezensis*, designated as *B. velezensis* BN, was screened from *Bacillus* strains previously isolated in our laboratory. The strain *B. velezensis* BN was isolated from healthy bulbs of *Lilium brownii* (lily) collected from Lintao County, Gansu Province, and exhibits strong broad-spectrum antifungal activity (Zheng et al., 2025). Through optimization strategies involving single-factor experiments and response surface methodologies, the surfactin yield was enhanced to 5.70 g L^−1^, representing a 5.94-fold increase compared to the initial level. This enhanced surfactin yield was accompanied by improved antifungal activity, biofilm formation, and rhizosphere colonization of *B. velezensis* BN. These findings provide a robust empirical foundation for advancing the application of surfactin in PGPR.

## 2 Materials and methods

### 2.1 Strains and culture conditions

*Bacillus subtilis* KC (New Yangshao, CN), *Bacillus laterosporus* CB (YIHAO, CN), *Bacillus megaterium* AA (JIWEI, CN), *Bacillus cereus* LY (WANGFA, CN), *Paenibacillus polymyxa* YF (NCBI: MW205750) (Zhang et al., 2023), *Bacillus velezensis* BN (NCBI: OR995192.1) (Zheng et al., 2025), and *Bacillus amyloliquefaciens* HT (NCBI: MW776428) (Lan et al., 2024) were obtained in previous laboratory studies. The plasmid pGFP4412 utilized in this study was sourced from Fenghui Biotech.

The media and their respective compositions utilized in this study are presented in Table 1.

**Table 1:** Composition of various media for microbial culture.

Medium name	Composition
Lysogeny broth (LB)	Bacto-tryptone 10.0 g L^−1^, yeast extract 5.0 g L^−1^, NaCl 10.0 g L^−1^
Nutrient broth (NB)	Peptone 10.0 g L^−1^, beef extract 3.0 g L^−1^, NaCl 5.0 g L^−1^, agar 20.0 g L^−1^, pH 7.3
Tryptic soy broth (TSB)	Tryptone 17.0 g L^−1^, soybean meal digest 3.0 g L^−1^, NaCl 5.0 g L^−1^, glucose 2.5 g L^−1^, pH 7.3
Landy broth	Glucose 20.0 g L^−1^, L-Glu 5.0 g L^−1^, KH₂PO₄ 1.0 g L^−1^, KCl 0.5 g L^−1^, MgSO₄·7H₂O 0.25 mg L^−1^, FeSO₄·7H₂O 0.15 mg L^−1^, MnSO₄·4H₂O 5.0 mg L^−1^, CuSO₄·5H₂O 0.16 mg L^−1^, agar 20.0 g L^−1^
Potato dextrose agar (PDA) medium	200 g of potatoes are boiled in 1 L of distilled water for 30 min, then filtered to obtain the extract. Glucose 20 g L^−1^, agar 15 g L^−1^, the volume was adjusted to 1 L with distilled water

The seed culture conditions: activated strains were streaked onto LB plates without antibiotics, and single colonies were picked and inoculated into LB liquid medium and cultured overnight at 30°C, 160 r min^−1^.

The fermentation conditions: in a 250 mL shaker flask containing 100 mL of fermentation medium, the seed culture was inoculated at a ratio of 5%, placed at 30°C and incubated at 160 r min^−1^ for 48 h.

### 2.2 Screening of high surfactin-producing strains

Surfactin quantification was performed using the cetylpyridinium chloride (CPC)-bromothymol blue (BTB) colorimetric assay as described by Yang et al. (2015). Briefly, the fermentation broths from the aforementioned different bacteria were collected at the end of fermentation, centrifuged at 8,000 × g for 10 min at room temperature to obtain the cell-free supernatants, which were subsequently stored at 4°C for the quantification of surfactin. Equal volumes of 0.1 M phosphate buffer saline (PBS) buffer (NaH₂PO₄/Na₂HPO₄ (XiLong, CN), pH 8.0), 0.2 mM CPC (JS Huayu, CN), and 0.2 mM BTB (Rhawn, CN) were mixed. An 800 μL aliquot of the mixture was combined with 100 μL of the cell-free supernatant in a 96-well microplate and allowed to react at room temperature for 5 min, each experimental group was performed in triplicate. The absorbance at 600 nm was measured using a microplate reader, and the strain with the highest absorbance value was selected for further experimental analysis. Statistical analysis was performed by one-way ANOVA with a Tukey test for multiple comparisons.

### 2.3 Preparation of lipopeptide crude extract

The preparation of lipopeptide crude extract was carried out based on the method described by Qian et al. (2022), with minor modifications. The sample pre-treatment method procedure was the consistent with that employed in CPC-BTB colorimetric assays. The supernatant was adjusted to pH 2.0 with 6 M HCl (Nanjing Reagent, CN) and incubated at 4°C overnight. Subsequently, the mixture was centrifuged at 5,000 × g for 10 min at 4°C, discarding the supernatant, and the precipitate was extracted using methanol (Aladdin, CN) at a volume three times that of the original supernatant, and the methanol-insoluble impurities were removed. The extract was concentrated by vacuum evaporation at 45°C to yield a light yellow lipopeptide crude extract, which was redissolved in 2 mL of methanol, and filtered through a 0.22 μm membrane for further analysis.

### 2.4 Quantification of surfactin content using high-performance liquid chromatography

The surfactin standard (Macklin, CN) was dissolved in methanol to prepare a stock solution at a concentration of 10 g L^−1^. This solution was subsequently diluted to concentrations of 2 g L^−1^, 4 g L^−1^, 6 g L^−1^, 8 g L^−1^, 10 g L^−1^ to establish a standard curve. The quantification of surfactin was performed using high-performance liquid chromatography (HPLC) (Shimadzu, Japan) on an XDB-C18 column (5 μm, 150 × 4.6 mm, Shimadzu, Japan).

Chromatography was performed at 40°C. Mobile phases of water with 0.1% (v/v) TFA (A) and methanol with 0.1% (v/v) TFA (B) were used, flow rate was set at 1.0 mL min^−1^, eluted with a solvent composition of A:B = 1:9 (v/v) over a total run time of 40 min. The capillary voltage was maintained at 3.5 kV, with ultraviolet detection set at a wavelength of 205 nm. A standard curve was generated by correlating the total peak area with the respective concentrations of the standards. The surfactin content in the lipopeptide crude extract from *B. velezensis* BN was quantified by referencing the peak area against the standard curve.

### 2.5 Optimization of surfactin yield

To determine the optimal basal medium for *B. velezensis* BN, three commonly used bacterial culture media [LB (Bertani, 1951), TSB (Yu and Fein, 2017), and NA (Rath et al., 2018)] and one surfactin-producing media [Landy (Akpa et al., 2001)] were selected. The seed culture of *B. velezensis* BN was inoculated at 5% (v/v) and fermented at 30°C and 160 r min^−1^ for 48 h. The medium with the highest biomass, measured as OD_600_, was selected as the basis for further optimization of culture conditions.

To identify the nutrients that enhance bacterial biomass and surfactin production when added to the culture medium, single-factor experiments were conducted. Each of the single factors was individually introduced into the basal LB medium, followed by a comparative analysis of surfactin yield between the medium with the added single factor and the control basal LB medium. Common carbon sources, including glucose (XiLong, CN), sucrose (Biosharp, CN), fructose (DAMAO, CN), amylum (FUCHEN, CN), and maltose (ZhongQin, CN), were selected as exogenous carbon sources and one of them was added at a concentration of 10 g L^−1^. Beef extracts (AOBOX, CN), carbamide (DAMAO, CN), and (NH_4_)_2_NO_3_ (Biosharp, CN) were chosen as exogenous nitrogen sources and one of them was added at a concentration of 5 g L^−1^. Amino acids, which are essential precursors for surfactin synthesis, were also evaluated. Surfactin is composed of β-hydroxy fatty acids and a cyclic peptide chain, with the latter consisting of five α-amino acids: L-Asp, L-Leu, L-Val, L-Glu, and D-Leu (Qi et al., 2023). These amino acids (all from Macklin, CN) were added exogenously at a concentration of 6 g L^−1^. Metal ions are essential nutrients for the normal metabolic activities of microorganisms and play a significant regulatory role in microbial metabolism. Previous studies have demonstrated that the concentration of metal ions significantly affects the production of lipopeptide biosurfactants (Arutchelvi et al., 2014; Bartal et al., 2018; Yang et al., 2020). Therefore, Fe^2+^ (FeSO_4_·7H_2_O, XiLong, CN), Mn^2+^ (MnSO_4_·H_2_O, BAISHI, CN), Mg^2+^ (MgSO_4_·7H_2_O, XiLong, CN), and K^+^ (KCl, XIHUA, CN) were selected as exogenous metal ions and added at a concentration of 0.05 M. Additionally, the influence of culture conditions on surfactin production was assessed by screening initial pH (5, 6, 7, and 8) and temperature (27°C, 30°C, 33°C, and 36°C) to determine the optimal conditions. In particular, three factors with the most pronounced influence on surfactin yield were identified and prioritized for subsequent optimization. Statistical analysis was performed using a one-way analysis of variance (ANOVA) with a Tukey test for multiple comparisons.

To further enhance surfactin yield, a Box–Behnken design (BBD) was implemented using Design-Expert software to evaluate the interactive effects of the principal component factors identified in the single-factor optimization experiments. To determine the central level of the model, a steepest ascent experiment was conducted with the three most significant factors. Specifically, beef extract concentration was increased by 5 g L^−1^ per step, while L-Asp and L-Leu concentrations were incremented by 3 g L^−1^ per step. Each treatment was replicated in triplicate. The experimental design of the steepest ascent experiment is detailed in Table 1. The optimal conditions obtained from the steepest ascent experiment were used as the central level of the model. These factors were treated as independent variables, with their experimental levels coded as −1, 0, and +1. The factors and levels used in the experimental protocol are presented in Table 2. The quantification of surfactin in each experiment was performed as described in the previous section.

**Table 2:** Test conditions for the steepest climbing path of the three most influential factors.

Run order	Factors			Yield of surfactin (g L^−1^)
	Beef extract (g L^−1^)	L-Leu (g L^−1^)	L-Asp (g L^−1^)	
1	25	7	7	4.67
2	30	10	10	5.05
3	35	13	13	5.45
4	40	16	16	5.66
5	45	19	19	5.32

As shown in the table, the surfactin yield reached its highest level in the fourth treatment group, which can be used as the midpoint for the experiment.

### 2.6 Determination of antifungal activity

To evaluate the antifungal activity of the sterile filtrate of *B. velezensis* BN against multiple fungal pathogens, a co-cultivation assay was conducted using petri dishes containing the sterile culture filtrate of *B. velezensis* BN, as previously described by Lin et al. (2024). The study encompassed four distinct fungi: *Colletotrichum gloeosporioides*, *Fusarium oxysporum*, *Alternaria alternata*, and *Phytophthora infestans*. *B. velezensis* BN was incubated under both optimized (Modified LB medium containing 40.11 g L^−1^ beef extract, 16.37 g L^−1^ L-Asp, and 16.06 g L^−1^ L-Leu, adjusted to pH 7.0, was incubated at 33°C.) and unoptimized (LB medium, pH 7.5, at 30°C) culture conditions for 48 h. The fermentation broth was then filtered through a 0.22 μm sterile membrane to obtain the sterile filtrate, which was incorporated into the PDA medium at two different ratios, resulting in final filtrate concentrations of 40 and 20% in the PDA plates. Control plates were devoid of the sterile filtrate.

After a 7-day incubation period, the pathogens were inoculated at the center of the PDA plates. Each experiment was repeated three times to ensure statistical robustness. All plates were incubated at 28°C until the fungal growth in the control plates reached the edges, at which point the colony radii were measured to calculate the inhibition rate. The fungal growth inhibition rate was calculated using the following formula:


$$\begin{array}{l} \text{Antifungal rate}\;\left(\%\right)=\\ \;\frac{\left(R_{\text{Colony of control group}}-R_{\text{Colony of treatment group}}\right)}{R_{\text{Colony of control group}}}\;\times 100\% \end{array}$$

### 2.7 Surfactin concentration and antifungal activity correlation

To elucidate the correlation between the antimicrobial activity of *B. velezensis* BN sterile filtrate and the surfactin concentration, toxicity assays were performed against four pathogenic fungi on PDA plates supplemented with surfactin (Macklin, CN) at different concentrations. The final concentrations of surfactin in the PDA plates were 40 μg mL^−1^ and 20 μg mL^−1^. The fungal cultivation procedures were conducted as previously described. The radial growth of fungal colonies was then measured, each experimental condition was subjected to triplicate analysis to ensure data reliability.

### 2.8 Crystal violet staining of biofilm formation

This study included two experimental groups: a control group (Unopt) utilizing LB medium and a treatment group (Opt) using an optimized medium (Opt medium). Following the method described by Mhatre et al. (2016), activated *B. velezensis* BN was inoculated at a 1% (v/v) into both LB and Opt medium. The cultures were then dispensed into a 6-well cell culture plate, with 2 mL of medium per well. Each well contained a sterile coverslip, and the plates were incubated statically at 33°C. After 48 h, the biofilm formation at the air–liquid interface was assessed. The quantification of biofilm formation was conducted using the crystal violet (CV) staining method, as previously described by Zheng et al. (2023). CV (Chinook, United States), an alkaline dye, binds to cellular components within the biofilm. Biofilm biomass was subsequently quantified through destaining and measurement of optical density. Statistical analysis was performed by one-way ANOVA with a Tukey test for multiple comparisons.

### 2.9 Potato seedling cultivation and inoculation

Through preliminary experiments, we demonstrated that *B. velezensis* BN exhibited significant inhibitory activity against *Phytophthora infestans*, the pathogen responsible for potato late blight. Based on these findings, we chose potato (*Solanum tuberosum* L.) as a model plant for pot cultivation studies. Following the protocol described by Tian et al. (2022), the Atlantic variety of potatoes from Ronghui market, Anning district, Lanzhou city, Gansu province, was selected, healthy potato tubers were washed thoroughly and surface sterilized. The potatoes were then sectioned into bud-eye-containing pieces using sterile surgical scissors and planted in pots filled with sterilized soil, with three seed pieces per pot. Potato plants were cultivated in a greenhouse under a temperature regime of 25°C/15°C (day/night, 16 h/8 h) and a relative humidity of 60–80%, with irrigation applied every 3 days. After sprouting, potato plants were further cultivated for 15 days, after which the intact root systems were carefully uprooted and the root surfaces thoroughly cleaned to remove adhering soil.

The potato plants were allocated into three experimental groups, each comprising three replicate pots: a control group treated with sterile LB medium, an unoptimized group treated with *B. velezensis* BN fermentation broth cultured in LB medium, and an optimized group treated with *B. velezensis* BN fermentation broth cultured in Opt medium. The roots of the treatment group were submerged in a 1-L beaker containing 500 mL of *B. velezensis* BN fermentation broth at a concentration of 1.0 × 10^−8^ CFU mL^−1^ for 3 h to promote bacterial adhesion. The roots of control group were immersed in sterile LB medium for the same duration to serve as a comparative baseline. The environmental conditions were maintained consistent with those of the previous cultivation. Subsequently, the roots of potato plants in the optimized group were immersed in sterile Opt culture medium diluted 100-fold with sterile water, while those in the non-optimized group were immersed in sterile LB medium similarly diluted 100-fold with sterile water. Both sets of samples were placed in separate beakers and incubated at 25°C in a greenhouse for 48 h to evaluate the adhesion capability of BN on the root surface under different culture conditions.

### 2.10 Scanning electron microscopy analysis

Biofilm samples were prepared for SEM analysis (Zeiss Ultra Plus, Germany) using a modified protocol based on that described by Fuchs et al. (2018). The method for biofilm cultivation was performed as described in section 2.8. The upper culture medium was aspirated and the biofilms were gently washed with sterile 0.01 M PBS to remove unattached bacteria. Subsequently, 2.5% glutaraldehyde (JYS, Wuhan, CN), pre-cooled to 4°C, was added and the samples were fixed overnight at 4°C. The glutaraldehyde was then aspirated, and the biofilms were washed three times with sterile 0.01 M PBS. Dehydration was performed using a graded ethanol series (30, 50, 70, 80, 90, and 100%) for 15 min per concentration. The 6-well plates were transferred to a vacuum freeze-dryer and dried for 24 h. The dried biofilms were carefully removed along with the cover glass, and the morphology and thickness of the *B. velezensis* BN biofilms in different media were examined using scanning electron microscopy (SEM). Each sample was analyzed in triplicate.

For SEM analysis of bacterial attachment on plant roots, root samples were prepared according to the method described by Zhai et al. (2023), with minor modifications. Root tissues, approximately 0.5 cm in length and located near the root tip, were excised using a sterile scalpel. The samples were fixed overnight in a 2.5% glutaraldehyde solution (prepared in 0.1 M phosphate buffer, pH 7.4) pre-cooled to 4°C. After fixation, the samples were washed three times with sterile 0.01 M PBS for 15 min each. Subsequently, the samples underwent ethanol dehydration in a graded series (50, 70, 80, 90, 95, and 100%) for 15 min per step. The dehydrated samples were then subjected to freeze-drying using a vacuum freeze-dryer. SEM analysis was performed on the potato root systems of different treatment groups, as described above, with each group being replicated three times.

### 2.11 Confocal laser scanning microscopy analysis

To quantitatively assess the rhizosphere colonization of potato roots by *B. velezensis* BN, the method described by He et al. (2021) was employed. The main principle of this method relies on the use of confocal laser scanning microscopy (CLSM) to visualize the colonization patterns of GFP-labeled bacterial strains. *B. velezensis* BN was genetically modified to carry the pGFP4412 plasmid, which encodes green fluorescent protein (GFP) and confers ampicillin (Amp) resistance, resulting in the GFP-labeled strain GFP-BN. The GFP protein enables spontaneous green fluorescence, allowing the visualization of bacterial colonization on potato roots under CLSM.

Five-week-old potato plants with surface-sterilized roots were transplanted into sterilized soil and divided into three groups for irrigation: a control group, a group inoculated with GFP-BN cultured in unoptimized LB medium, and a group inoculated with GFP-BN cultured in Opt medium. Both treatment groups received 100 mL of a GFP-BN suspension at a concentration of 1.0 × 10^8^ CFU mL^−1^, while the control group was irrigated with sterile LB medium. The potato plants were cultivated in a greenhouse under the same conditions as described above for 10 days, with irrigation using sterile water every 3 days. Each treatment was replicated three times. After harvesting, roots were rinsed, sectioned into 1 mm slices, and analyzed using CLSM (Olympus FV3000, Japan) with excitation at 488 nm.

### 2.12 Quantification of GFP-BN colonization on potato roots

To quantify the colonization of GFP-BN on potato roots, 1.0 g of root tissue was homogenized in a mortar with 9.0 mL of sterile distilled water to prepare a uniform suspension. This suspension was serially diluted and plated onto modified LB medium supplemented with Amp (100 μg mL^−1^). Following incubation at 30°C for 24 h, colony counts were recorded to determine the colonization level of GFP-BN on the roots. Statistical analysis was performed using a one-way ANOVA with a Tukey test for multiple comparisons.

### 2.13 Statistical analysis

Data were analyzed using one-way Analysis of Variance (ANOVA) to determine the significance of differences among groups. The ANOVA test was performed using SPSS software (version 23.0, IBM Corp., Armonk, NY, United States). When the ANOVA indicated a significant effect (*p <* 0.05), the Duncan’s multiple range test was applied *post hoc* to identify which groups differed significantly from each other. The results are presented with different lowercase letters (e.g., a, b, c) to denote significant differences among the groups. If the means of two groups are followed by different letters, it indicates that the difference between them is statistically significant at the *p <* 0.05 level.

## 3 Results

### 3.1 Screening for strain with high surfactin yield and identifying the surfactin produced

**Figure 1:**
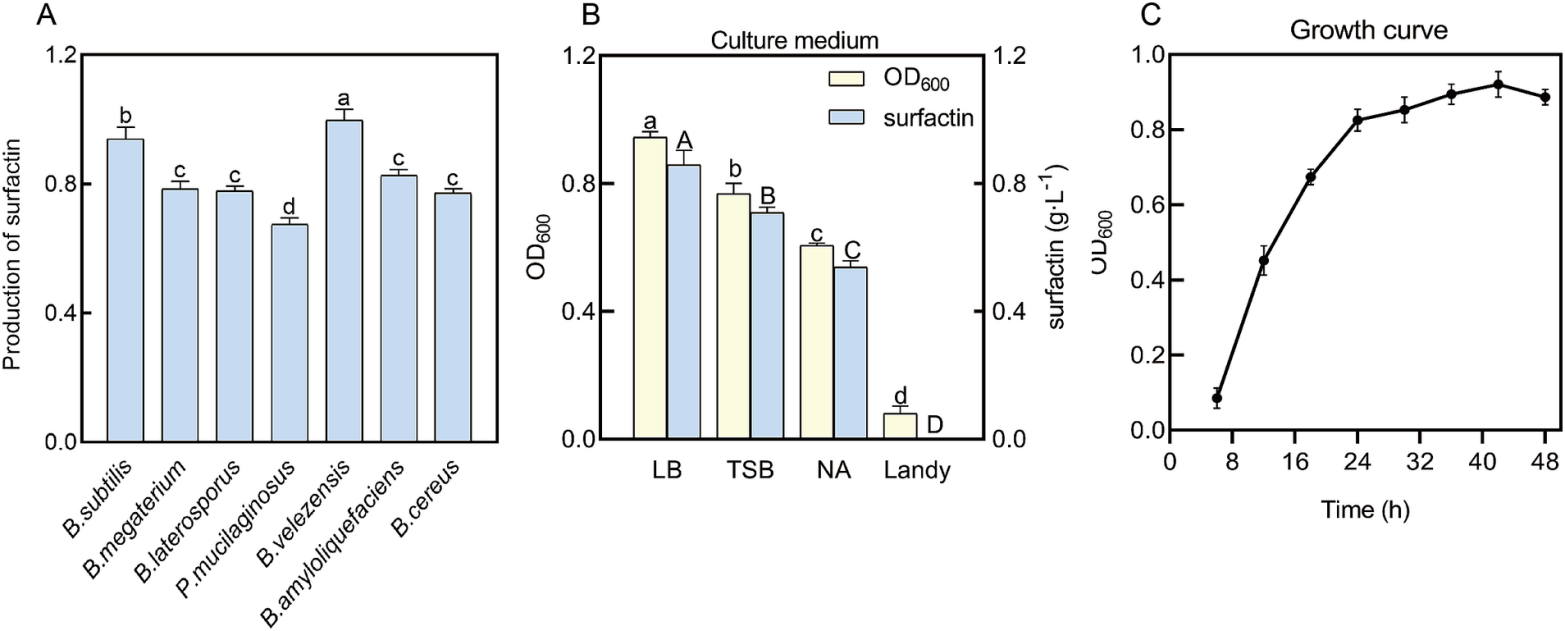
Determination of experimental strains and basic culture conditions. **(A)** Comparison of surfactin yields among different PGPR strains using the CPC-BTB colorimetric assay. **(B)** Screening for the optimal culture medium. **(C)** Growth curve of *B. velezensis* BN. Error bars indicate standard deviations of results from three biological replications. Different lowercase letters on the column indicated significant differences in analysis (*p* < 0.05). *B. subtilis* (*Bacillus subtilis*), *B. megaterium* (*Bacillus megaterium*), *B. laterosporus* (*Bacillus laterosporus*), *P. polymyxa* (*Paenibacillus polymyxa*), *B. velezensis* (*Bacillus velezensis*), *B. amyloliquefaciens* (*Bacillus amyloliquefaciens*), and *B. cereus* (*Bacillus cereus*).

To identify strains with the highest surfactin yield, several common PGPR strains were screened using the CPC-BTB assay. A surfactin standard at 5 mg mL^−1^ was used for calibration. As illustrated in Figure 1A, *B. velezensis* BN demonstrated the highest surfactin yield and was subsequently selected for further optimization experiments.

Due to the sensitivity and accuracy limitations of the CPC-BTB assay, HPLC method was established for precise quantification. Calibration curves were generated using standards, and the retention time was determined (Supplementary Figure 1). Surfactin are composed of multiple homologs, typically ranging from C_12_ to C_17_, with their chromatographic retention times exhibiting a linear relationship with the number of carbon atoms in the fatty acid chain (Yu et al., 2024). The HPLC chromatogram of the lipopeptide crude extract from *B. velezensis* BN showed distinct peaks corresponding to the retention times of various surfactin homologs, confirming the presence of multiple variants in the lysate.

### 3.2 Influence of nutritional and environmental factors on surfactin yield

The population density of bacteria cultures is a pivotal factor influencing antimicrobial activity and colonization efficacy (Li et al., 2022). Since the bacterial concentration in liquid culture is proportional to optical density at 600 nm within a specific range, various standard media were evaluated for their impact on the OD_600_ values of *B. velezensis* BN. As shown in Figure 1B, LB medium produced the highest OD_600_ values, followed by NA and TSB media, while growth in Landy medium was markedly inhibited. Since the culture conditions, including temperature and pH, were maintained consistently across all media, the observed growth inhibition in Landy medium is presumably due to specific components within the medium that negatively impacted the growth of *B. velezensis* BN. Consequently, LB medium was selected as the basal medium for further optimization. The growth curve of *B. velezensis* BN (Figure 1C) demonstrated that the strain reached the logarithmic growth phase at 24 h. As cellular metabolism is most active during this phase (Corbet, 1933), cultures harvested at 24 h were utilized as the inoculum for subsequent experiments. Surfactin, a type of secondary metabolite, typically reach peak secretion during the stationary phase (Hoff et al., 2021). Therefore, the fermentation supernatant was harvested after 48 h to maximize surfactin recovery.

**Figure 2:**
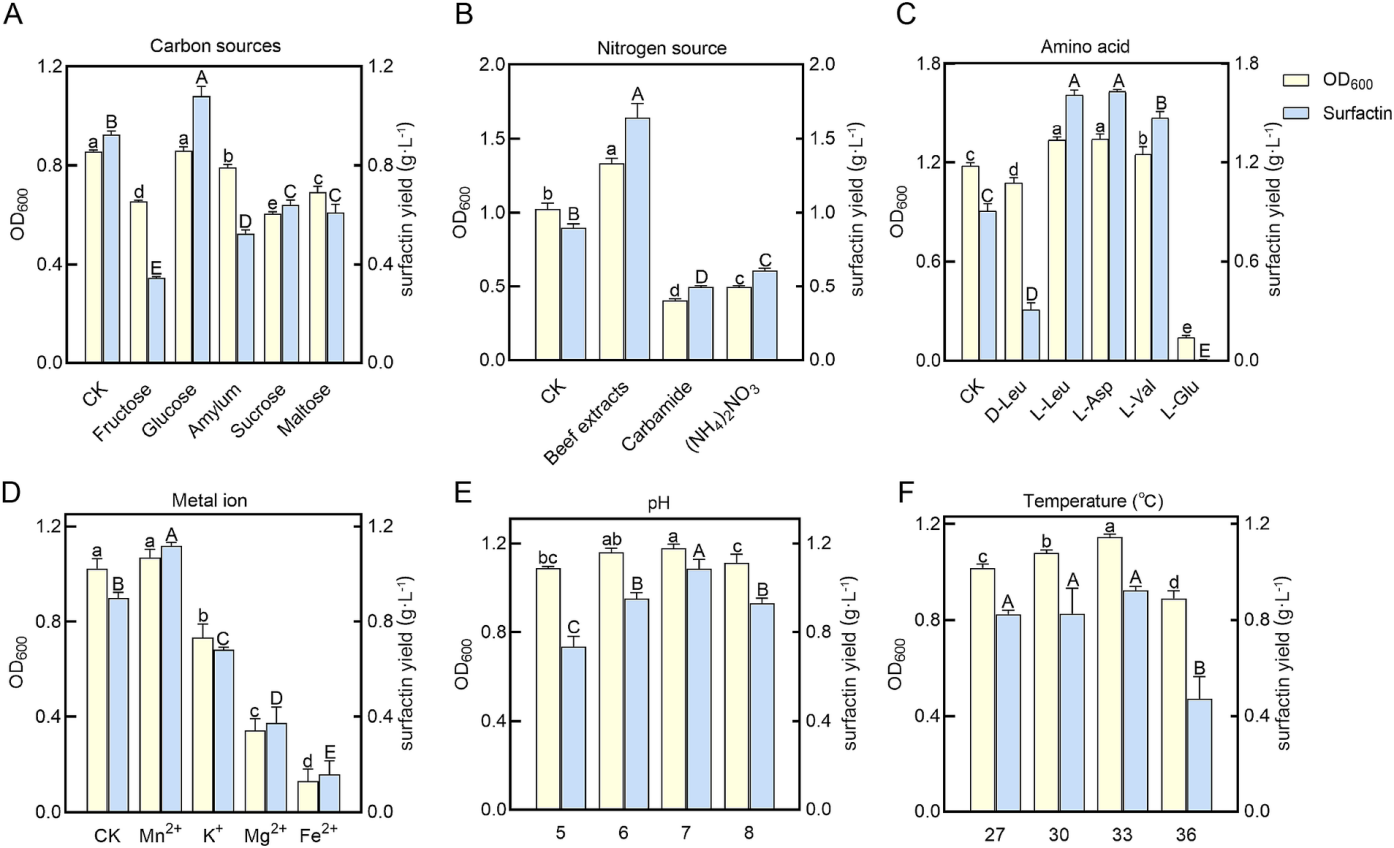
Measurement of bacterial biomass (OD_600_) and surfactin yields in *B. velezensis* BN under varying nutritional and environmental conditions. HPLC was used to analyze and compare the production of surfactin under various single-factor conditions. **(A)** Carbon sources. **(B)** Nitrogen sources. **(C)** Amino acids. **(D)** Metal ions. **(E)** pH. **(F)** Temperatures. Error bars indicate standard deviations of results from three biological replications. The distinct letters superimposed on the bars signify statistically significant differences (*p <* 0.05) as determined by the analysis. The lowercase letters are utilized to denote variations in bacterial biomass, whereas the uppercase letters correspond to disparities in surfactin yield levels.

Under the initial fermentation conditions, the surfactin yield in the supernatant of *B. velezensis* BN was 0.97 ± 0.08 g L^−1^. Single-factor optimization experiments were conducted to investigate the effects of individual nutritional factors and environmental conditions on surfactin yield and biomass of *B. velezensis* BN, with results shown in Figure 2. Among carbon sources, glucose proved optimal, enhancing surfactin yield by 1.13-fold compared to the basal medium while supporting maximum biomass. Beef extract, as a nitrogen source, resulted in a 1.61-fold increase in surfactin yield. Analysis of amino acids revealed that L-Leu and L-Asp significantly stimulated surfactin synthesis, with increases of 1.41-fold and 1.40-fold, respectively, compared to the control. The addition of Mn^2+^ further elevated surfactin yield to 1.22-fold the control level. The optimal growth temperature for *B. velezensis* BN was determined to be 33°C, consistent with typical growth ranges for *Bacillus* sp. (25–35°C) (Luo et al., 2023). When the initial pH was set at 7.0, both biomass and surfactin yield reached their maximum levels. Therefore, single-factor optimization indicated that the concentrations of beef extract, L-Asp, and L-Leu were the most influential factors.

To further enhance surfactin yield, response surface methodology (RSM) was employed, focusing on the three key variables identified in single-factor optimization. As shown in Table 1, treatment group 4 achieved the highest surfactin yield, 5.66 ± 0.03 g L^−1^, suggesting that the factor levels in this group were near-optimal and were used as the central point for the central composite design.

**Figure 3:**
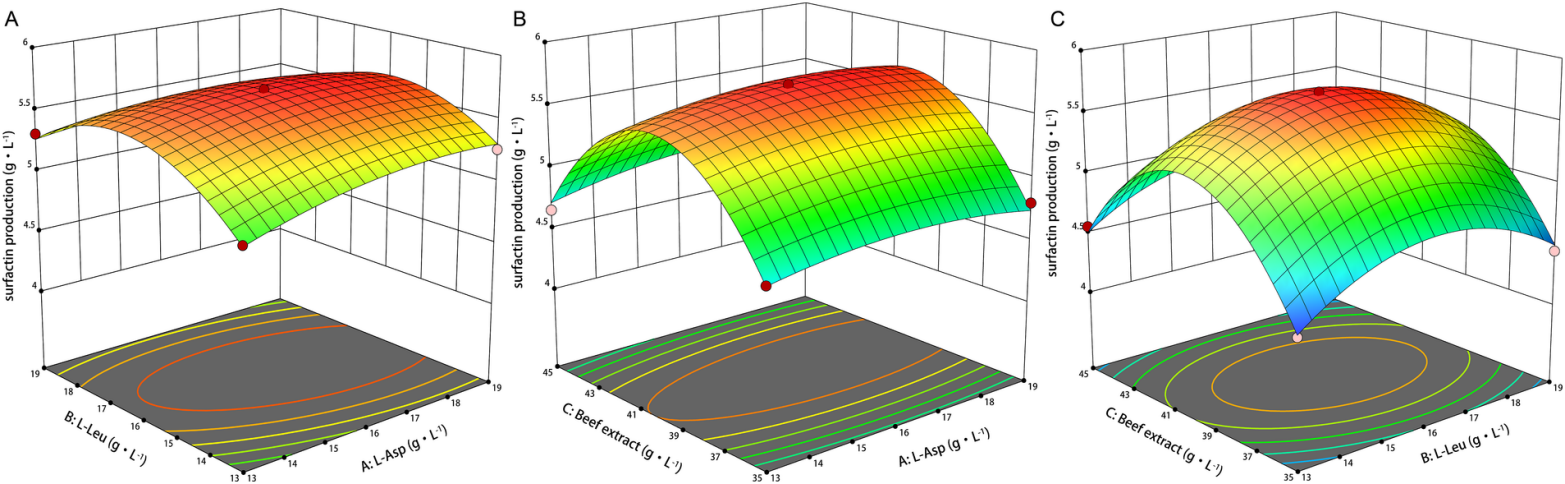
Response surface plots of L-Asp, L-Leu, and beef extract on surfactin yields. **(A)** Response surface plot of L-Asp and L-Leu. **(B)** Response surface plot of L-Asp and beef extrac. **(C)** Response surface plot of L-Leu and beef extract.

Design-Expert 19 software was used to develop the experimental design, with factor levels and responses provided in Supplementary Table 1. The 3D response surface plots in Figures 3A–C illustrate the effects of beef extract, L-Asp, and L-Leu on surfactin yield. Analysis of variance (ANOVA) (Table 3) confirmed the statistical significance of the model (*p <* 0.05), with coefficients of determination (*R*^2^ and *R*_adj_^2^) exceeding 0.9 and a nonsignificant lack of fit, demonstrating high reliability and predictive accuracy (see Table 4).

**Table 3:** Response surface experimental factors and levels.

Code	Factors	Level		
		−1	0	1
X_1_	L-Asp (g L^−1^)	13	16	19
X_2_	L-Leu (g L^−1^)	13	16	19
X_3_	Beef extract (g L^−1^)	35	40	45

**Table 4:** Analysis of variance for the multiple quadratic regression model.

Source	Sum of squares	df	Mean square	*F*-value	*p*-value	Significance
Model	3.18	9	0.3537	82.06	0.0004	*
A-L-Asp	0.0211	1	0.0211	4.89	0.0914	
B-L-Leu	0.0081	1	0.0081	1.88	0.2422	
C-Beef extract	0.0114	1	0.0114	2.64	0.1797	
AB	0.0341	1	0.0341	7.91	0.0482	*
AC	0.0053	1	0.0053	1.23	0.3290	
BC	0.0031	1	0.0031	0.7166	0.4449	
A^2^	0.1423	1	0.1423	33.00	0.0046	*
B^2^	0.8053	1	0.8053	186.83	0.0002	*
C^2^	2.77	1	2.77	641.61	<0.0001	**
Residual	0.0172	4	0.0043			
Lack of fit	0.0167	3	0.0056	10.40	0.2232	
Pure error	0.0005	1	0.0005			

*R*_Adj_^2^ = 0.9828; * indicates significant difference (*p* < 0.05); ** indicates extremely significant difference (*p* < 0.01).

The regression for surfactin yield was determined as follows:


$$\begin{array}{l} Y=0.0513\;X_{1}+0.0318\;X_{2}+0.0377\;X_{3}-0.0923\;X_{1}X_{2}\\ \;+0.0365\;X_{1}X_{3}-0.0278\;X_{2}X_{3}-0.2058\;{X_{1}}^{2}-0.4967\;{X_{2}}^{2}\\ \;-0.9246\;{X_{3}}^{2}+5.69 \end{array}$$

where Y represents surfactin yield (g L^−1^), and X_1_, X_2_, and X_3_ are the concentrations of beef extract (g L^−1^), L-Asp (g L^−1^), and L-Leu (g L^−1^), respectively.

The model predicted a maximum surfactin yield of 5.698 g L^−1^ under the following conditions: 40.11 g L^−1^ beef extract, 16.37 g L^−1^ L-Asp, and 16.06 g L^−1^ L-Leu, at pH 7.0, 33°C, and 160 r min^−1^. Experimental validation confirmed an actual maximum surfactin yield of 5.70 g L^−1^, demonstrating a 99.8% correlation with the predicted value, and showing an increase of 5.94-fold compared to the initial level.

### 3.3 Surfactin exhibits antagonistic activity against various plant pathogenic fungi

**Figure 4:**
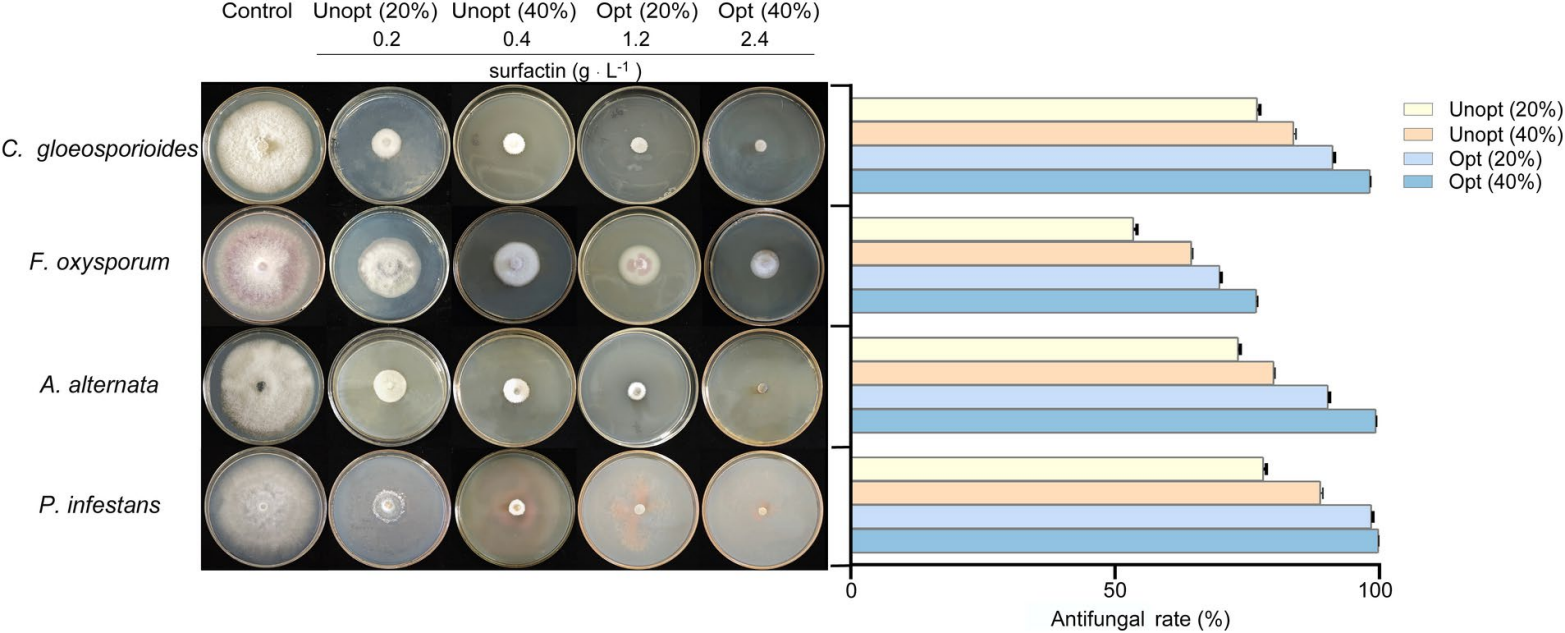
Antagonistic effects (left) and statistical analysis (right) against four pathogenic fungi in sterile filtrates containing different concentrations. Error bars indicate standard deviations of results from three biological replications. *C. gloeosporioides* (*Colletotrichum gloeosporioides*), *F. oxysporum* (*Fusarium oxysporum*), *A. alternata* (*Alternaria alternata*), and *P. infestans* (*Phytophthora infestans*).

The antifungal efficacy of surfactin against various plant pathogens was assessed using inhibition assays on potato dextrose agar (PDA) plates supplemented with sterile filtrates of *B. velezensis* BN containing varying surfactin concentrations. The results demonstrated that the fermentation supernatant of *B. velezensis* BN exhibited significant inhibitory effects on the tested plant pathogens (Figure 4). Notably, the inhibitory activity intensified as surfactin concentrations in the fermentation supernatant increased.

**Figure 5:**
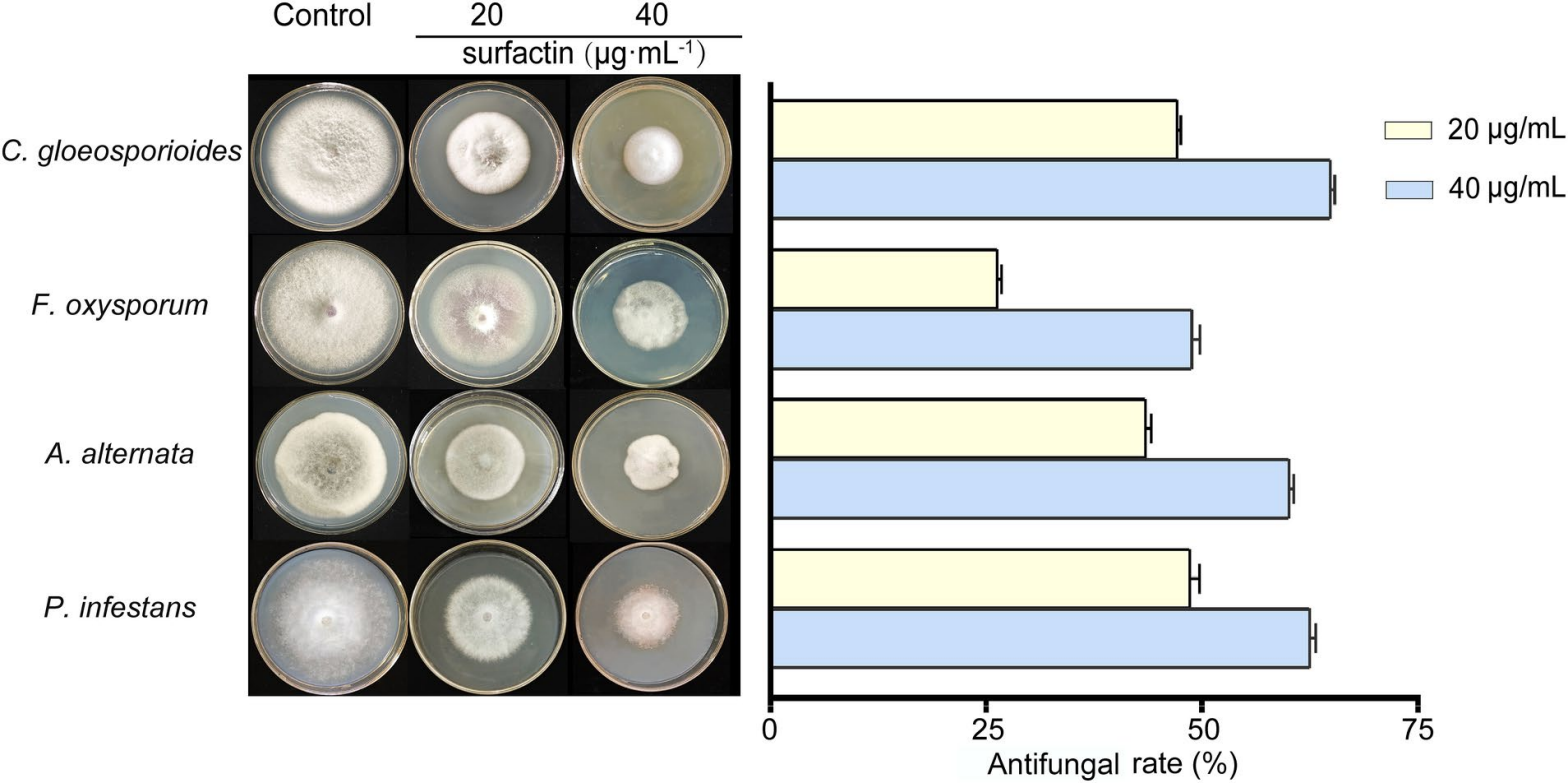
Antagonistic effects (left) and statistical analysis (right) of inhibition rates against four pathogenic fungi using PDA plates with surfactin concentrations of 20 μg mL^−1^ and 40 μg mL^−1^. Error bars indicate standard deviations of results from three biological replications. *C. gloeosporioides* (*Colletotrichum gloeosporioides*), *F. oxysporum* (*Fusarium oxysporum*), *A. alternata* (*Alternaria alternata*), and *P. infestans* (*Phytophthora infestans*).

Among the tested pathogens, *P. infestans* was the most susceptible to the sterile filtrate. At a 20% sterile filtrate concentration, the inhibition rate for the unoptimized group was 78.06%, whereas the optimized group achieved an enhanced inhibition rate of 98.47%, representing a 20.41% improvement. Similarly, the optimized group showed increased inhibitory effects against *C. gloeosporioides*, *A. alternata*, and *F. oxysporum*, with inhibition rates rising from 76.91, 73.31, and 53.52% in the unoptimized group to 91.17, 90.31, and 69.80%, respectively (Figure 4 right). At a higher concentration (40%) of sterile filtrate, the unoptimized group displayed inhibition rates of 83.77, 64.46, 80.02, and 88.84% against *C. gloeosporioides*, *F. oxysporum*, *A. alternata*, and *P. infestans*, respectively. In contrast, the optimized group exhibited a remarkable rise to 98.23, 76.73, 99.35, and 99.97%, respectively. These results indicate that as the concentration of sterile filtrate increases, so does the antagonism activity against fungi. As the concentration of sterile filtrate increases, so does the concentration of surfactin present in the agar plates. To further confirm the relationship between surfactin concentration and antifungal activity, additional experiments were conducted using PDA plates directly supplemented with the surfactin standard (Figure 5). Surfactin at a concentration of 20 μg mL^−1^ exhibited inhibition rates of 47.21, 26.35, 43.54, and 48.67% against *C. gloeosporioides*, *F. oxysporum*, *A. alternata*, and *P. infestans*, respectively. Increasing the surfactin concentration to 40 μg mL^−1^ resulted in higher inhibition rates of 64.91, 48.88, 60.16, and 62.53% against the respective pathogens. These findings highlight a consistent increase in antifungal activity with elevated surfactin concentrations, demonstrating a direct correlation between surfactin levels and their antifungal potency within the tested concentration range.

### 3.4 Biofilm and colonization density of *Bacillus velezensis* BN

**Figure 6:**
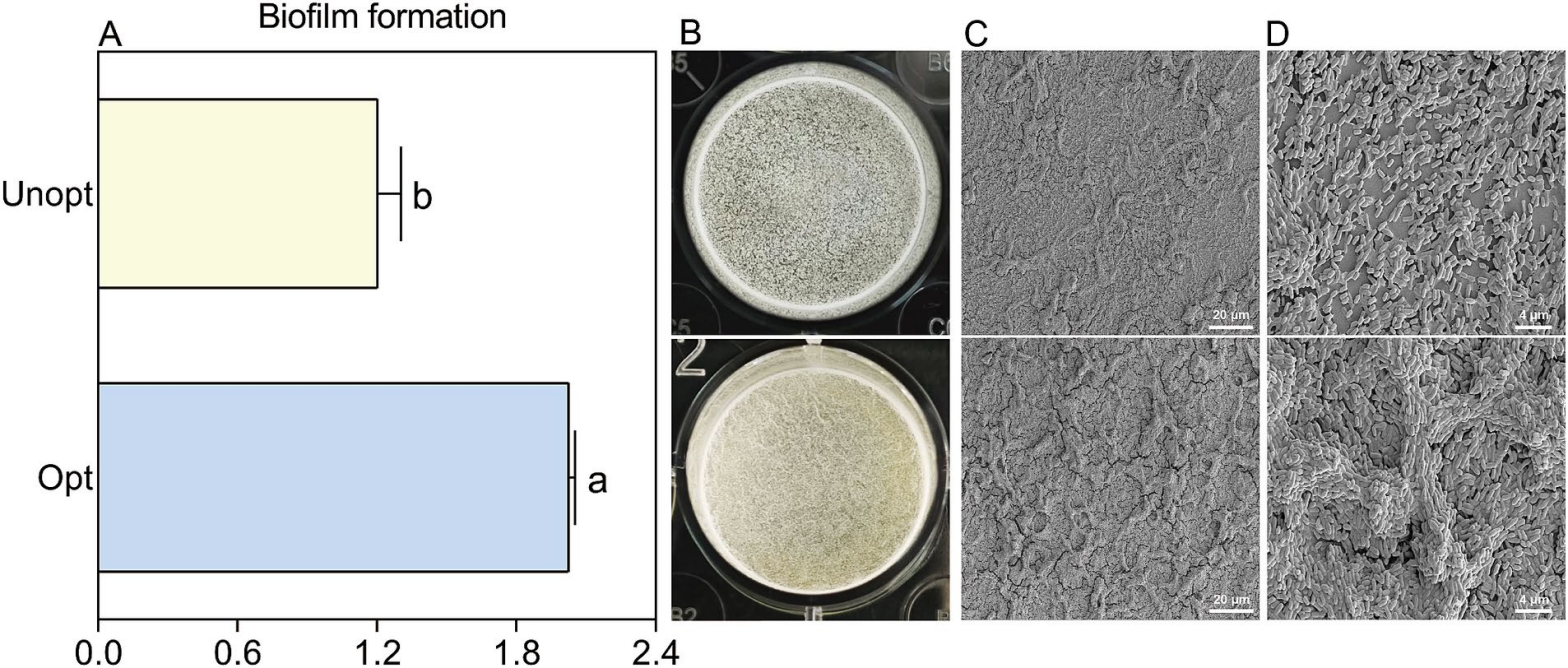
The morphology sturcture and formation of *B. velezensis* BN biofilms under optimized and unoptimized conditions. Error bars indicate standard deviations of results from three biological replications. Different lowercase letters on the column indicated significant differences in analysis (*p <* 0.05). **(A)** Quantification of biofilm formation using CV staining. **(B)** Direct observation of the biofilm morphology. **(C)** Morphological observation of biofilms under SEM at 300× magnification. **(D)** Morphological observation of biofilms under SEM at 2,000× magnification.

The biofilm-forming capacity of *B. velezensis* BN, a critical factor for rhizosphere colonization, was assessed to investigate the influence of surfactin yield on bacterial colonization potential. The results of the crystal violet staining further corroborated these findings, showing a 68.6% increase in biofilm formation in the optimized medium compared to the unoptimized control (Figure 6A). This enhancement in biofilm formation was also notably evident in the optimized group, as confirmed by the distinct, wrinkled biofilm layer observed at the liquid-air interface (Figure 6B). SEM analysis at 300× magnification (Figure 6C) demonstrated a denser bacterial population and more pronounced biofilm wrinkling in the optimized group. Additional SEM images at 2,000× magnification (Figure 6D) highlighted clear differences in biofilm morphology between the two groups. These results collectively indicate that increased surfactin yield significantly enhances biofilm formation in *B. velezensis* BN.

**Figure 7:**
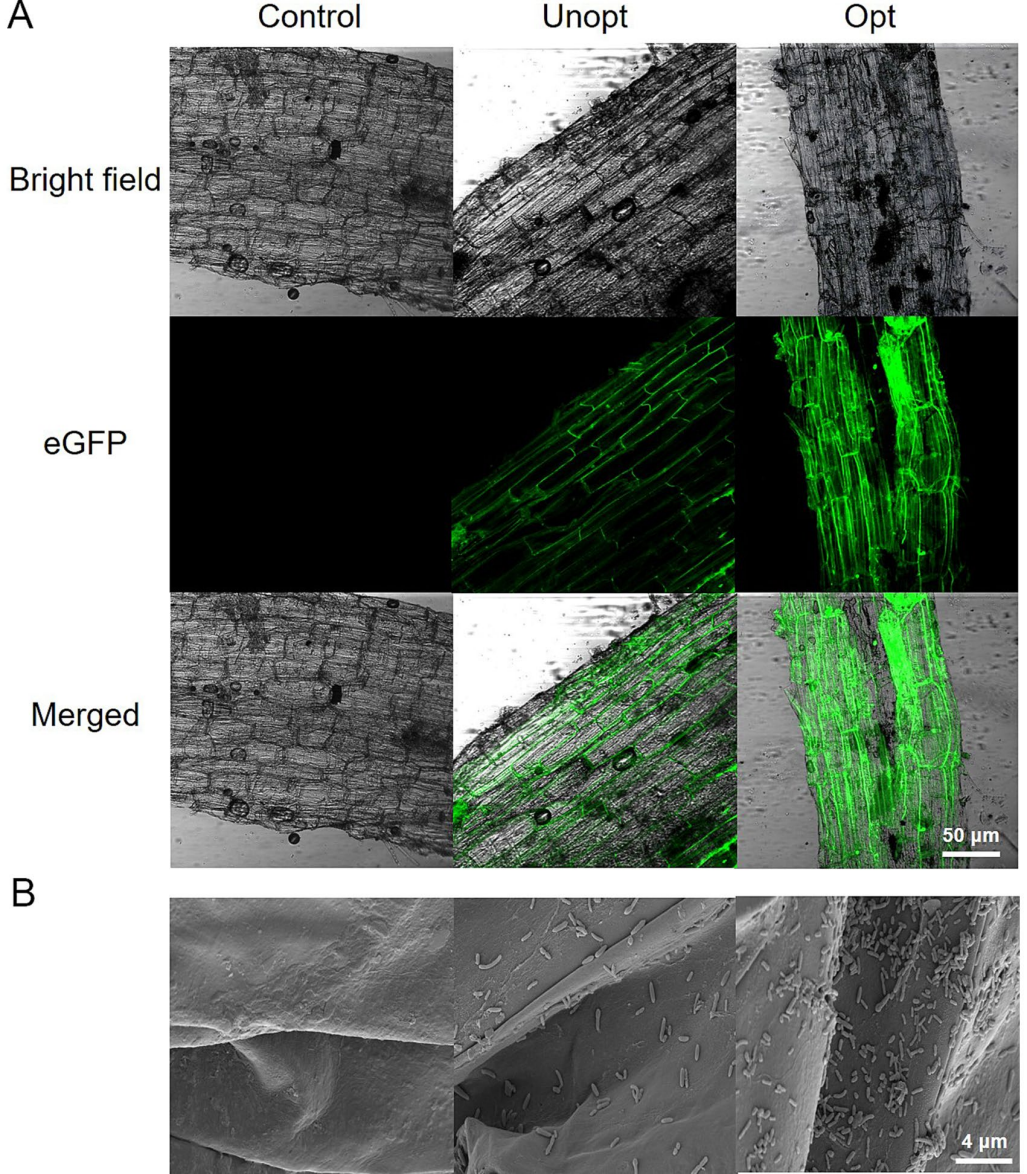
Colonization of *B. velezensis* BN on potato roots under optimized and unoptimized conditions. **(A)** Analysis of GFP-BN colonization on potato roots using CLSM. **(B)** Analysis of attachment dynamics of *B. velezensis* BN on potato root surfaces using SEM.

**Figure 8:**
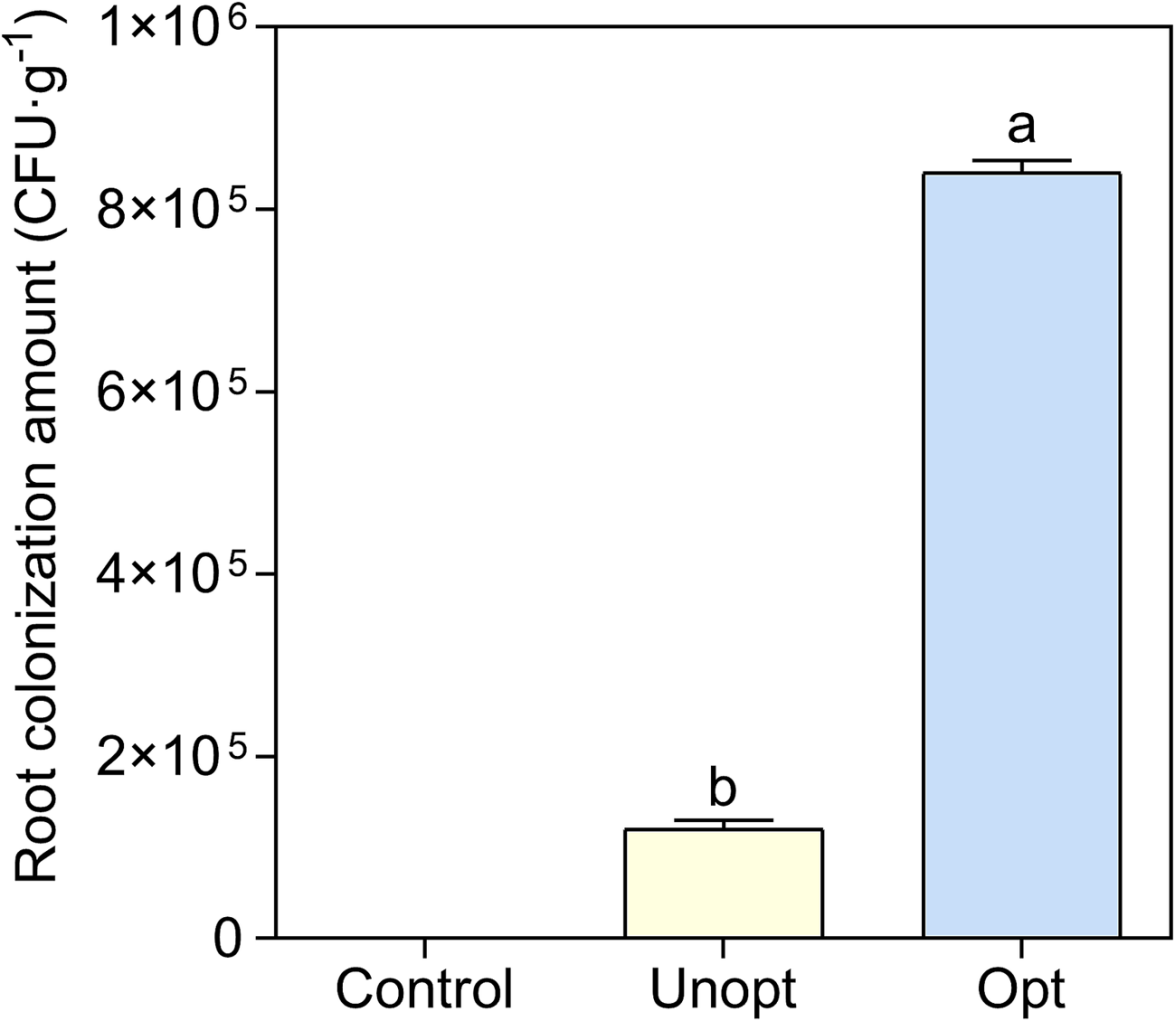
Statistical analysis of GFP-BN colony counts under various cultivation conditions. Error bars indicate standard deviations of results from three biological replications. Different lowercase letters on the column indicated significant differences in analysis (*p* < 0.05).

The colonization capacity of *B. velezensis* BN on potato roots was further evaluated under optimized and unoptimized conditions. CLSM analysis (Figure 7A) revealed minimal green fluorescence in the unoptimized group, while the optimized group exhibited bright fluorescence, indicating enhanced colonization of GFP-tagged *B. velezensis* BN under optimized conditions. SEM imaging (Figure 7B) further confirmed these observations, with the unoptimized group displayed sparse, scattered bacterial cells on root surfaces, whereas the optimized group exhibited robust bacterial adhesion, suggesting significantly improved attachment capacity under optimized conditions. Colony-forming unit (CFU) enumeration from potato root homogenates (Figure 8) showed that the colonization density in the optimized group reached 9.4 × 10^5^ CFU g^−1^, a 7.83-fold increase compared to 1.2 × 10^5^ CFU g^−1^ in the unoptimized group.

## 4 Discussion

Surfactin is composed of a peptide chain and β-hydroxy fatty acids through an ester bond. Its unique amphiphilic structure can disrupt the permeability and integrity of pathogen cell membranes, thereby inhibiting their growth and reproduction. The biosynthesis of fatty acid, especially branched-chain fatty acid, is crucial for surfactin yield (Xia et al., 2024). Pyruvate generated during glycolysis is catalyzed by pyruvate dehydrogenase (*phdABCD*) to form acetyl-CoA, marking the initiation of branched-chain fatty acid synthesis. The increased yield of surfactin by *B. velezensis* BN upon glucose supplementation in the medium may be attributed to elevated intracellular pyruvate levels resulting from the increased glucose concentration in the medium (Figure 2A) (Chen et al., 2020).

Surfactin, a cyclic lipopeptide, consists of a peptide chain and β-hydroxy fatty acids. Branched-chain amino acids, including L-Val, L-Leu, and L-Ile, are essential for surfactin synthesis. These amino acids function both as precursors for the surfactin peptide chain and as regulators of microbial metabolic pathways, thereby significantly influencing surfactin yield (Yu et al., 2024). Supplementation of L-Asp and L-Leu in the cultivation medium significantly enhanced surfactin yield, indicating their positive regulatory roles in surfactin biosynthesis (Figure 2C). Notably, the supplement of L-Leu has been reported to boost surfactin yield by up to 20.9-fold (Coutte et al., 2015). Consistent with this, Wang et al. (2020) demonstrated that the addition of L-Leu during the shake flask fermentation of the *B. subtilis* THBS-2 strain resulted in approximately a twofold increase in surfactin yield. In addition, our study revealed that L-Glu nearly arrested the growth of *B. velezensis* BN, suggesting a negative regulatory effect of environmental L-Glu on its proliferation. In section 3.2, we discussed the growth inhibition of *B. velezensis* BN observed in Landy medium, which is likely due to specific components within the medium that adversely affect its growth. Considering that L-Glu is one of the primary constituents of Landy medium, we hypothesize that L-Glu could be the principal factor contributing to the growth inhibition of *B. velezensis* BN in this medium. While D-Leu supplementation did not affect bacterial growth, it significantly reduced surfactin yield (Figure 2C), likely due to substrate competition with endogenous D-Leu transpeptidase, which may disrupt enzymatic activity and hinder surfactin biosynthesis (Zhou et al., 2019). The research conducted by Du et al. (2023) revealed that the supplementation of D-Leu markedly decreased the production of surfactin, with the yield dropping from an initial concentration of 2.25 g L^−1^ to 0.2 g L^−1^.

The hydrophobic domain of surfactin integrates into the lipid bilayer, while its hydrophilic segment remains exposed to the extracellular environment. This insertion disrupts the integrity of membrane by increasing permeability, ultimately leading to structural damage (Chen et al., 2022). Such disruption interferes with normal cellular processes, resulting in the death of microbial cells and achieving effective antifungal activity (Falk, 2019). Furthermore, Fan et al. (2017) demonstrated that surfactin is the primary antimicrobial agent produced by *B. subtilis* 9407, as mutants deficient in surfactin synthesis exhibited minimal inhibitory effects on fungal growth. This study evaluated the growth inhibition of various pathogenic fungi on PDA plates supplemented with different concentrations of sterile filtrates derived from *B. velezensis* BN fermentation. The findings revealed that higher concentrations of sterile filtrate corresponded to increased inhibition rates against the fungi. However, this effect was not strictly linear, possibly due to the presence of other inhibitory components in the filtrate. To further elucidate the relationship between surfactin concentration and antifungal activity, additional tests were performed on PDA plates containing varying concentrations of purified surfactin. The results were consistent with those obtained using sterile filtrates, confirming that fungal inhibition rates increased with higher surfactin concentrations, thus establishing a positive correlation between surfactin levels and antifungal efficacy (Figure 5).

Biofilm formation and root colonization are closely interconnected in *Bacillus* spp., with surfactin playing a pivotal role in these processes. Surfactin reduces surface tension and acts as a lubricant, thereby facilitating the adhesion and aggregation of bacteria on host surfaces—prerequisites essential for biofilm establishment. Under adverse environments conditions, surfactin can also trigger biofilm formation by initiating signaling pathways (Gough, 2009). Biofilms consist primarily of extracellular polysaccharides (EPS) and specific proteins such as TasA, which confers structural stability, and BslA, which introduces hydrophobic properties to the biofilm matrix. The genetic regulation of these components involves the *epsA-O* operon, the *tapA-sipW-tasA* operon, and the *bslA* gene. Surfactin-induced signaling can activate the global regulator Spo0A (Townsley et al., 2018), which subsequently regulates the expression of these operons, thereby promoting biofilm formation. Zhang et al. (2022) demonstrated that Spo0A is essential for biofilm formation in *B. velezensis* R9, *B. licheniformis* WH1, *B. cereus* 285-3, and *B. subtilis* CYY, with the strongest effect observed in *B. velezensis* R9. Similarly, our findings indicate that surfactin significantly contributes to biofilm formation in *B. velezensis* BN (Figure 6).

In addition, this study explored the effect of surfactin concentration on root colonization by *B. velezensis* BN, revealing a positive correlation between surfactin levels and the colonization capacity of *B. velezensis* BN. Colonization assays demonstrated that *B. velezensis* BN exhibited enhanced root colonization when cultivated in surfactin-optimized media compared to LB broth (Figure 7). Consistent with these findings, Al-Ali et al. (2017) reported that wild-type *B. velezensis* could form robust biofilms and stably colonize in the roots of tomatoes, whereas surfactin-deficient mutants failed to colonize. Fan et al. (2017) showed that the mutant lacking surfactin biosynthesis exhibited significantly reduced biofilm formation and motility, both of which were restored by exogenous surfactin supplementation. These findings underscore the pivotal role of surfactin in biofilm formation and root colonization by *B. velezensis*. Future research could employ genetic engineering techniques such as CRISPR/Cas9 to modify key regulatory genes, enhancing biofilm stability and production in *B. velezensis* BN.

This study optimized the cultivation conditions of *B. velezensis* BN to enhance surfactin yield, examining its impact on the antifungal activity and colonization efficiency. Our results demonstrate that the addition of L-leucine, L-aspartate, and beef extract significantly increased surfactin yield to 5.70 g L^−1^, this optimized medium thus provides a scientific basis for improving surfactin yield. Future research could build on the optimized medium developed in this study by using it as a baseline for a series of new studies. These studies could explore the potential of additional medium components, such as glucose, L-Val, and Mn^2+^, identified in this study as potentially promising additives. This approach may lead to further improvements in surfactin yield, contributing to the development of more effective biopesticides.

Our results underscore the crucial role of surfactin in the biocontrol capabilities of PGPR. By improving surfactin yield, this study provides a scientific basis for the development of biopesticides, thereby advancing sustainable agriculture. These findings contribute to a deeper understanding of surfactin-mediated biocontrol mechanisms, offering both theoretical foundations and technical support for the development of efficient biopesticides.

## Data Availability

The original contributions presented in the study are included in the article/Supplementary material, further inquiries can be directed to the corresponding author.

## References

[ref1] Akpa E. Jacques P. Wathelet B. Paquot M. Fuchs R. Budzikiewicz H.. (2001). Influence of culture conditions on lipopeptide production by *Bacillus subtilis*. Appl. Biochem. Biotechnol. 91, 551–561. doi: 10.1385/abab:91-93:1-9:551 11963884

[ref2] Al-Ali A. Deravel J. Krier F. Béchet M. Jacques P. (2017). Biofilm formation is determinant in tomato rhizosphere colonization by *Bacillus velezensis* FZB42. Environ. Sci. Pollut. Res. Int. 25, 29910–29920. doi: 10.1007/s11356-017-0469-1 29063401

[ref3] Arutchelvi J. Sangeetha J. Philip J. Doble M. (2014). Self-assembly of surfactin in aqueous solution: role of divalent counterions. Colloids Surf. B 116, 396–402. doi: 10.1016/j.colsurfb.2013.12.034 24524939

[ref4] Atwa N. A. El-Shatoury E. H. Elazzazy A. M. Abouzeid M. A. El-Diwany A. I. (2013). Enhancement of surfactin production by *Bacillus velezensis* NRC-1 strain using a modified bench-top bioreactor. J. Agric. Food Environ. 1111, 169–174.

[ref5] Babalola O. O. Akanmu A. O. Ayangbenro A. S. (2024). Draft genome sequence of *Bacillus velezensis* strains AOA1 and AKS2, the potential plant growth-promoting rhizobacteria. Microbiol. Resour. Announc. 13:e0087723. doi: 10.1128/mra.00877-23 38411072 PMC11008119

[ref6] Bartal A. Vigneshwari A. Bóka B. Vörös M. Takács I. Kredics L.. (2018). Effects of different cultivation parameters on the production of surfactin variants by a *Bacillus subtilis* strain. Molecules 23:2675. doi: 10.3390/molecules23102675, PMID: 30340314 PMC6222309

[ref7] Bertani G. (1951). Studies on lysogenesis. I. The mode of phage liberation by lysogenic *Escherichia coli*. J. Bacteriol. 62, 293–300. doi: 10.1128/jb.62.3.293-300.1951, PMID: 14888646 PMC386127

[ref8] Chen X. Lu Y. Shan M. Zhao H. Lu Z. Lu Y. (2022). A mini-review: mechanism of antimicrobial action and application of surfactin. World J. Microbiol. Biotechnol. 38:143. doi: 10.1007/s11274-022-03323-3, PMID: 35718798

[ref9] Chen B. Wen J. Zhao X. Ding J. Qi G. (2020). Surfactin: a quorum-sensing signal molecule to relieve CCR in *Bacillus amyloliquefaciens*. Front. Microbiol. 11:631. doi: 10.3389/fmicb.2020.00631 32425896 PMC7203447

[ref10] Corbet A. S. (1933). The bacterial growth curve and the history of species. Nature 131, 61–62. doi: 10.1038/131061a0

[ref11] Coutte F. Niehren J. Dhali D. John M. Jacques P. (2015). Modeling leucine’s metabolic pathway and knockout prediction improving the production of surfactin, a biosurfactant from *Bacillus subtilis*. Biotechnol. J. 10, 1216–1234. doi: 10.1002/biot.201400541, PMID: 26220295

[ref12] Dai C. Yan P. Yin X. Shu Z. Mintah B. K. He R.. (2025). Surfactin and its antibacterial mechanism on staphylococcus aureus and application in pork preservation. Food Bioprocess Technol. 18, 1311–1324. doi: 10.1007/s11947-024-03528-4

[ref13] Das B. B. Dkhar M. S. (2011). Rhizosphere microbial populations and physico chemical properties as affected by organic and inorganic farming practices. Am. Eurasian J. Agric. Environ. Sci. 10, 140–150.

[ref14] Deravel J. Lemière S. Coutte F. Krier F. Hese N. V. Béchet M.. (2014). Mycosubtilin and surfactin are efficient, low ecotoxicity molecules for the biocontrol of lettuce downy mildew. Appl. Microbiol. Biotechnol. 98, 6255–6264. doi: 10.1007/s00253-014-5663-1, PMID: 24723290

[ref15] Dong L. Wang P. Zhao W. Su Z. Zhang X. Lu X.. (2022). Surfactin and fengycin contribute differentially to the biological activity of *Bacillus subtilis* NCD-2 against cotton verticillium wilt. Biol. Control 174:104999. doi: 10.1016/j.biocontrol.2022.104999

[ref16] Du Y. Wang Y. Cui T. Ge L. Yu F. Zhao M.. (2023). Efficient production of biosurfactant surfactin by a newly isolated *Bacillus subtilis* (sp.) 50499 strain from oil-contaminated soil. Food Bioprod. Process. 142, 40–49. doi: 10.1016/j.fbp.2023.09.002

[ref17] Falk N. A. (2019). Surfactants as antimicrobials: a brief overview of microbial interfacial chemistry and surfactant antimicrobial activity. J. Surfactant Deterg. 22, 1119–1127. doi: 10.1002/jsde.12293, PMID: 32336911 PMC7166552

[ref18] Fan H. Zhang Z. Li Y. Zhang X. Duan Y. Wang Q. (2017). Biocontrol of bacterial fruit blotch by *Bacillus subtilis* 9407 via surfactin-mediated antibacterial activity and colonization. Front. Microbiol. 8:1973. doi: 10.3389/fmicb.2017.01973 29075242 PMC5641556

[ref19] Fuchs F. M. Gudrun H. Ralf M. Michael L. (2018). Directed freeze-fracturing of *Bacillus subtilis* biofilms for conventional scanning electron microscopy. J. Microbiol. Methods 152, 165–172. doi: 10.1016/j.mimet.2018.08.005 30125587

[ref20] Gough N. R. (2009). Ionic signals to community formation. Sci. Signal. 2:ec10. doi: 10.1126/scisignal.253ec10

[ref21] He P. Li S. Xu S. Zheng S. J. (2021). Monitoring tritrophic biocontrol interactions between *Bacillus* spp., *Fusarium oxysporum* f. sp. cubense, tropical race 4, and banana plants *in vivo* based on fluorescent transformation system. Front. Microbiol. 12:754918. doi: 10.3389/fmicb.2021.754918 34721361 PMC8550332

[ref22] Hezakiel H. E. Thampi M. Rebello S. Sheikhmoideen J. M. (2024). Biopesticides: a green approach towards agricultural pests. Appl. Biochem. Biotechnol. 196, 5533–5562. doi: 10.1007/s12010-023-04765-7, PMID: 37994977

[ref23] Hoff G. Arguelles Arias A. Boubsi F. Pršić J. Meyer T. Ibrahim H. M. M.. (2021). Surfactin stimulated by pectin molecular patterns and root exudates acts as a key driver of the *Bacillus*-plant mutualistic interaction. mBio 12, e01774–e01721. doi: 10.1128/mBio.01774-21, PMID: 34724831 PMC8561381

[ref24] Hu F. Liu Y. Li S. (2019). Rational strain improvement for surfactin production: enhancing the yield and generating novel structures. Microb. Cell Factories 18:42. doi: 10.1186/s12934-019-1089-x, PMID: 30819187 PMC6394072

[ref25] Kim K. M. Lee J. Y. Kim C. K. Kang J. S. (2009). Isolation and characterization of surfactin produced by *Bacillus polyfermenticus* KJS-2. Arch. Pharm. Res. 32, 711–715. doi: 10.1007/s12272-009-1509-2, PMID: 19471885

[ref26] Kinsinger R. F. Shirk M. C. Fall R. (2003). Rapid surface motility in *Bacillus subtilis* is dependent on extracellular surfactin and potassium ion. J. Bacteriol. 185, 5627–5631. doi: 10.1128/JB.185.18.5627-5631.2003, PMID: 12949115 PMC193742

[ref27] Kondo T. Sibponkrung S. Hironao K. Y. Tittabutr P. Boonkerd N. Ishikawa S.. (2023). *Bacillus velezensis* S141, a soybean growth-promoting bacterium, hydrolyzes isoflavone glycosides into aglycones. J. Gen. Appl. Microbiol. 69, 175–183. doi: 10.2323/jgam.2023.02.002 36858546

[ref28] Krishnan N. Velramar B. Velu R. K. (2019). Investigation of antifungal activity of surfactin against mycotoxigenic phytopathogenic fungus *Fusarium moniliforme* and its impact in seed germination and mycotoxicosis. Pestic. Biochem. Physiol. 155, 101–107. doi: 10.1016/j.pestbp.2019.01.010 30857619

[ref29] Lan Q. Liu Y. Mu R. Wang X. Zhou Q. Islam R.. (2024). Biological control effect of antagonistic bacteria on potato black scurf disease caused by *Rhizoctonia solani*. Agronomy 14:351. doi: 10.3390/agronomy14020351

[ref30] Li Q. Xing D. Xiao Y. Liao S. Zou Y. Liu F.. (2022). Rhizosphere colonization of *Bacillus subtilis* biocontrol strain SEM-9 and the effect on microbial diversity in rhizosphere soil. J. South China Agric. Univ. 43, 82–88. doi: 10.7671/j.issn.1001-411X.202107008

[ref31] Lin G. Sina F. Marten Abrimiti E. Macao H. Jing G. Boru Z. (2024). Identification of antagonistic bacteria against poplar skin rot and study on the antibacterial effect of sterile filtrate. J. Shandong For. Sci. Technol. 54, 1–7. doi: 10.3969/j.issn.1002-2724.2024.03.002

[ref32] Luo K. Chen Y. Qian X. Zhong H. Onchari M. M. Liu X.. (2023). Enhancing surfactin production in *B. velezensis* Bs916 combined cumulative mutagenesis and expression key enzymes. Appl. Microbiol. Biotechnol. 107, 4233–4244. doi: 10.1007/s00253-023-12590-5, PMID: 37231158

[ref33] Mhatre E. Troszok A. Gallegos-Monterrosa R. Lindstädt S. Kovács Á. T. (2016). The impact of manganese on biofilm development of *Bacillus subtilis*. Microbiology 162, 1468–1478. doi: 10.1099/mic.0.000320, PMID: 27267987

[ref34] Munusamy S. Conde R. Bertrand B. Munoz-Garay C. (2020). Biophysical approaches for exploring lipopeptide-lipid interactions. Biochimie 170, 173–202. doi: 10.1016/j.biochi.2020.01.009 31978418 PMC7116911

[ref35] Park G. Nam J. Kim J. Song J. Kim P. I. Min H. J.. (2019). Structure and mechanism of surfactin peptide from *Bacillus velezensis* antagonistic to fungi plant pathogens. Bull. Korean Chem. Soc. 40, 704–709. doi: 10.1002/bkcs.11757

[ref36] Qi X. Liu W. He X. Du C. (2023). A review on surfactin: molecular regulation of biosynthesis. Arch. Microbiol. 205:313. doi: 10.1007/s00203-023-03652-3, PMID: 37603063

[ref37] Qian R. Xu X. Xu Z. Xu H. Li S. Xu Z.. (2022). Isolation and identification of antibacterial lipopeptides from *Bacillus subtilis* KC-WQ fermentation broth and optimization of fermentation conditions. Sci. Technol. Food Ind. 43, 123–131. doi: 10.13386/j.issn1002-0306.2021100269

[ref38] Rath M. Mitchell T. R. Gold S. E. (2018). Volatiles produced by *Bacillus mojavensis* RRC101 act as plant growth modulators and are strongly culture-dependent. Microbiol. Res. 208, 76–84. doi: 10.1016/j.micres.2017.12.014 29551214

[ref39] Saleem S. Iqbal A. Ahmed F. Ahmad M. (2021). Phytobeneficial and salt stress mitigating efficacy of IAA producing salt tolerant strains in *Gossypium hirsutum*. Saudi J. Biol. Sci. 28, 5317–5324. doi: 10.1016/j.sjbs.2021.05.056, PMID: 34466110 PMC8381066

[ref40] Santoyo G. Urtis-Flores C. A. Loeza-Lara P. D. Orozco-Mosqueda M. D. C. Glick B. R. (2021). Rhizosphere colonization determinants by plant growth-promoting Rhizobacteria (PGPR). Biology 10:475. doi: 10.3390/biology10060475, PMID: 34072072 PMC8229920

[ref41] Stoll A. Salvatierra-Martínez R. González M. Araya M. (2021). The role of surfactin production by *Bacillus velezensis* on colonization, biofilm formation on tomato root and leaf surfaces and subsequent protection (ISR) against *Botrytis cinerea*. Microorganisms 9:2251. doi: 10.3390/microorganisms9112251, PMID: 34835375 PMC8626045

[ref42] Tian X. Zhao X. Zhao S. Zhao J. Mao Z. (2022). The biocontrol functions of *Bacillus velezensis* strain BV-25 against *Meloidogyne incognita*. Front. Microbiol. 13:843041. doi: 10.3389/fmicb.2022.843041 35464938 PMC9022661

[ref43] Townsley L. Yannarell S. M. Huynh T. N. Woodward J. J. Shank E. A. Ausubel F. M. (2018). Cyclic di-AMP acts as an extracellular signal that impacts *Bacillus subtilis* biofilm formation and plant attachment. mBio 9:e00341. doi: 10.1128/mBio.00341-18 29588402 PMC5874923

[ref44] Wang M. Yu H. Li X. Shen Z. (2020). Single-gene regulated non-spore-forming *Bacillus subtilis*: construction, transcriptome responses, and applications for producing enzymes and surfactin. Metab. Eng. 62, 235–248. doi: 10.1016/j.ymben.2020.08.008, PMID: 32835805

[ref45] Wu G. Yu Z. Ming W. (2024). The mechanism of PGPR regulating plant response to adversity stress. Acta Prataculturala Sin. 33, 203–218. doi: 10.11686/cyxb2023276

[ref46] Xia L. Hou Z. Zhu F. Wen J. (2024). Enhancing surfactin production in *Bacillus subtilis*: insights from proteomic analysis of nitrate-induced overproduction and strategies for combinatorial metabolic engineering. Bioresour. Technol. 397:130499. doi: 10.1016/j.biortech.2024.130499, PMID: 38417461

[ref47] Xiao P. Tian X. Zhu P. Xu Y. Zhou C. (2023). The use of surfactin in inhibiting *Botrytis cinerea* and in protecting winter jujube from the gray mold. AMB Express 13:37. doi: 10.1186/s13568-023-01543-w, PMID: 37118318 PMC10147881

[ref48] Yang N. Wu Q. Xu Y. (2020). Fe nanoparticles enhanced surfactin production in *Bacillus amyloliquefaciens*. ACS Omega 5, 6321–6329. doi: 10.1021/acsomega.9b03648, PMID: 32258866 PMC7114131

[ref49] Yang H. Yu H. Shen Z. (2015). A novel high-throughput and quantitative method based on visible color shifts for screening *Bacillus subtilis* Thy-15 for surfactin production. J. Ind. Microbiol. Biotechnol. 42, 1139–1147. doi: 10.1007/s10295-015-1635-4, PMID: 26065390

[ref50] Ye Q. Zhong Z. Chao S. Liu L. Chen M. Feng X.. (2023). Antifungal effect of *Bacillus velezensis* Zn-S10 against plant pathogen *Colletotrichum changpingense* and its inhibition mechanism. Int. J. Mol. Sci. 24:16694. doi: 10.3390/ijms242316694, PMID: 38069016 PMC10705930

[ref51] Yu Q. Fein J. B. (2017). Controls on bacterial cell envelope sulfhydryl site concentrations: the effect of glucose concentration during growth. Environ. Sci. Technol. 51, 7395–7402. doi: 10.1021/acs.est.7b01047, PMID: 28603975

[ref52] Yu F. Shen Y. Pang Y. Fan H. Liu M. Liu X. (2024). Effects of branched-chain amino acids on surfactin structure and antibacterial activity in *Bacillus velezensis* Ya215. World J. Microbiol. Biotechnol. 40, 1–14. doi: 10.1007/s11274-024-04088-7 39060617

[ref53] Zhai Q. Pan Z. Zhang C. Yu H. L. Zhang M. Gu X. H.. (2023). Colonization by *Klebsiella variicola* FH-1 stimulates soybean growth and alleviates the stress of *Sclerotinia sclerotiorum*. J. Integr. Agric. 22, 2729–2745. doi: 10.1016/j.jia.2023.01.007

[ref54] Zhang Y. Qi J. Wang Y. Wen J. Zhao X. Qi G. (2022). Comparative study of the role of surfactin-triggered signalling in biofilm formation among different *Bacillus* species. Microbiol. Res. 254:126920. doi: 10.1016/j.micres.2021.126920 34800863

[ref55] Zhang Z. Zhang W. Wang X. Kou Z. Wang Y. Islam R.. (2023). Isolation and identification of antagonistic bacteria of *Angelica* root rot and their mechanism as biological control. Biol. Control 177:105120. doi: 10.1016/j.biocontrol.2022.105120

[ref56] Zhang T. Zhou Q. (2022). Using large-scale multi-module NRPS to heterologously prepare highly efficient lipopeptide biosurfactants in recombinant *Escherichia coli*. Enzym. Microb. Technol. 159:110068. doi: 10.1016/j.enzmictec.2022.110068 35660853

[ref57] Zheng L. Gu X. Sun L. Dong M. Gao A. Han Z.. (2023). Adding metal ions to the *Bacillus mojavensis* D50 promotes biofilm formation and improves ability of biocontrol. J. Fungi 9:526. doi: 10.3390/jof9050526, PMID: 37233237 PMC10219225

[ref58] Zheng Y. Liu T. Wang Z. Wang X. Wang H. Li Y.. (2025). Whole-genome sequencing and secondary metabolite exploration of the novel *Bacillus velezensis* BN with broad-spectrum antagonistic activity against fungal plant pathogens. Front. Microbiol. 15:1498653. doi: 10.3389/fmicb.2024.1498653, PMID: 39831126 PMC11738913

[ref59] Zhou D. Hu F. Lin J. Wang W. Li S. (2019). Genome and transcriptome analysis of *Bacillus velezensis* BS-37, an efficient surfactin producer from glycerol, in response to D-/L-leucine. MicrobiologyOpen 8:e00794. doi: 10.1002/mbo3.794, PMID: 30793535 PMC6692528

