# Supplementary figures and images for: Optimizing surfactin yield in Bacillus velezensis BN to enhance biocontrol efficacy and rhizosphere colonization
Source: Front Microbiol. 2025 Mar 5;16:1551436. doi: 10.3389/fmicb.2025.1551436 (PMC11919844; doi:10.3389/fmicb.2025.1551436)

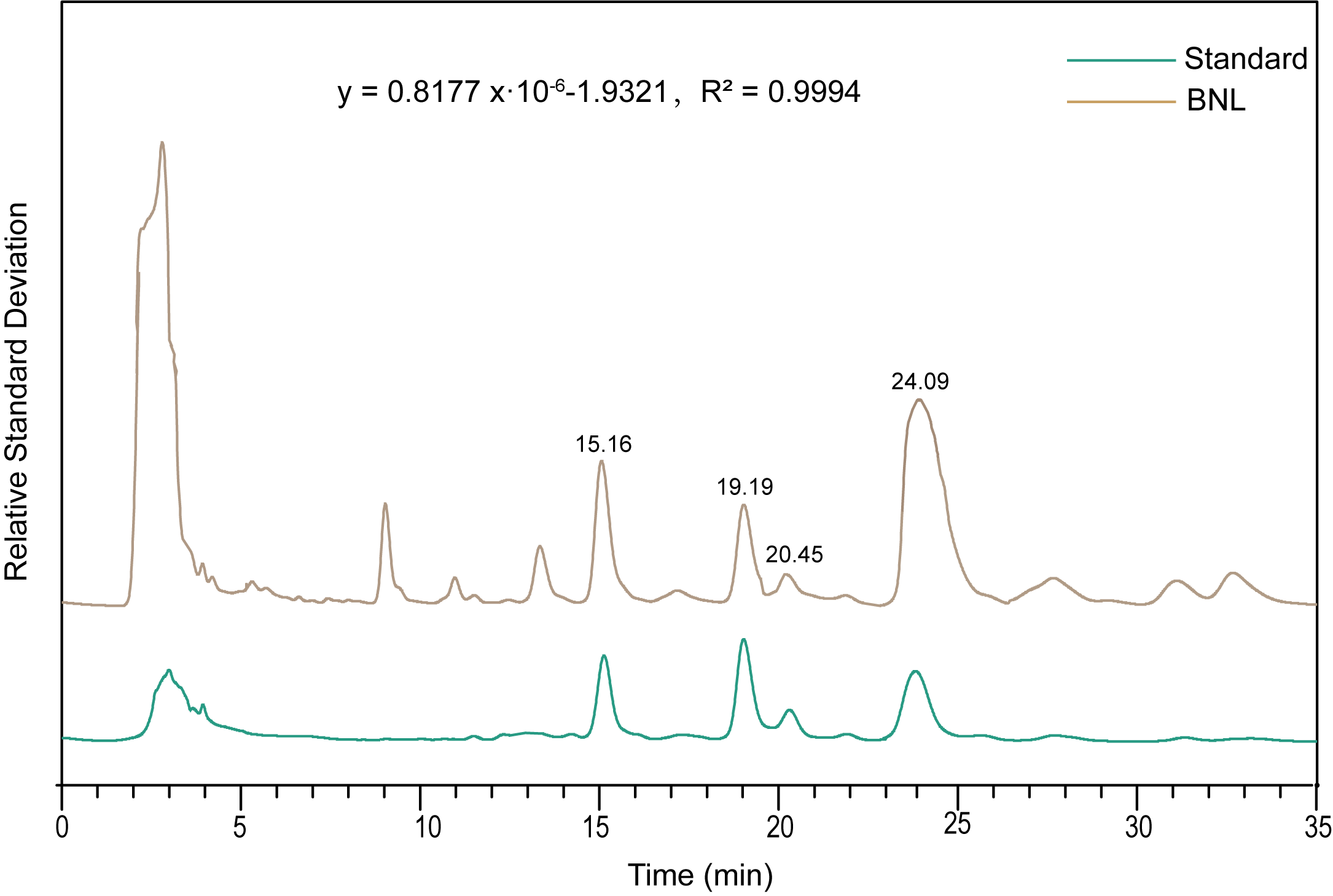

Supplement: Supplementary file 1 [file Image_1.tif]
