# Supplementary material for: Optimizing surfactin yield in Bacillus velezensis BN to enhance biocontrol efficacy and rhizosphere colonization
Source: Front Microbiol. 2025 Mar 5;16:1551436. doi: 10.3389/fmicb.2025.1551436 (PMC11919844; doi:10.3389/fmicb.2025.1551436)
Supplement: Supplementary file 2 [file Data_Sheet_1.docx]

**Supplementary Figures Legends:**

**Supplementary Figure 1**. Comparison of the liquid chromatography profiles of the crude lipopeptide extract from *B. velezensis* BN and the surfactin standard.

**Supplementary Table 1**: Response surface experimental design and measurement results.

| Run order | L-Asp  (g ∙ L^-1^) | L-Leu  (g ∙ L^-1^) | Beef extract  (g ∙ L^-1^) | surfactin production  (g ∙ L^-1^) |
| --- | --- | --- | --- | --- |
| 1 | 13 | 13 | 40 | 5.03±0.12 |
| 2 | 19 | 13 | 40 | 5.26±0.08 |
| 3 | 13 | 19 | 40 | 5.31±0.16 |
| 4 | 19 | 19 | 40 | 5.17±0.03 |
| 5 | 13 | 16 | 35 | 4.72±0.06 |
| 6 | 19 | 16 | 35 | 4.80±0.09 |
| 7 | 13 | 16 | 45 | 4.65±0.23 |
| 8 | 19 | 16 | 45 | 4.88±0.04 |
| 9 | 16 | 13 | 35 | 4.36±0.06 |
| 10 | 16 | 19 | 35 | 4.45±0.05 |
| 11 | 16 | 13 | 45 | 4.56±0.11 |
| 12 | 16 | 19 | 45 | 4.53±0.02 |
| 13 | 16 | 16 | 40 | 5.70±0.07 |
| 14 | 16 | 16 | 40 | 5.69±0.06 |
